# Exploring the eco-evolutionary role of plasmids and defense systems in ‘*Fervidacidithiobacillus caldus*’ extreme acidophile

**DOI:** 10.3389/fmicb.2025.1610279

**Published:** 2025-08-11

**Authors:** Sebastián Pacheco-Acosta, Gustavo Castro-Toro, Camila Rojas-Villalobos, Cesar Valenzuela, Juan José Haristoy, Abraham Zapata-Araya, Ana Moya-Beltrán, Pedro Sepúlveda-Rebolledo, Ernesto Pérez-Rueda, Ricardo Ulloa, Alejandra Giaveno, Francisco Issotta, Beatriz Díez, Simón Beard, Raquel Quatrini

**Affiliations:** ^1^Programa de Doctorado en Inmunología y Microbiología, Facultad de Medicina, Universidad San Sebastián, Santiago, Chile; ^2^Centro Científico y Tecnológico de Excelencia Ciencia & Vida, Santiago, Chile; ^3^Programa de Doctorado en Biología Computacional, Facultad de Ingeniería, Arquitectura y Diseño, Universidad San Sebastián, Santiago, Chile; ^4^Programa de Doctorado en Biotecnología y Bioemprendimiento, Facultad de Medicina, Universidad San Sebastián, Santiago, Chile; ^5^Departamento de Informática y Computación, Facultad de Ingeniería, Universidad Tecnológica Metropolitana, Santiago, Chile; ^6^Instituto de Investigaciones en Matemáticas Aplicadas y en Sistemas, Universidad Nacional Autónoma de México, Unidad Académica del Estado de Yucatán, Mérida, Yucatán, Mexico; ^7^PROBIEN (CCT Patagonia Confluencia-CONICET, UNCo), Facultad de Ingeniería, Departamento de Química, Universidad Nacional del Comahue, Neuquén, Argentina; ^8^Centro GEMA - Genómica, Ecología & Medio Ambiente, Universidad Mayor, Santiago, Chile; ^9^Millennium Institute Center for Genome Regulation (MI-CGR), Santiago, Chile; ^10^Center for Climate and Resilience Research (CR)2, Santiago, Chile; ^11^Departamento Genética Molecular y Microbiología, Facultad de Ciencias Biológicas, Pontificia Universidad Católica, Santiago, Chile; ^12^Facultad de Medicina, Universidad San Sebastián, Santiago, Chile; ^13^Facultad de Ciencias, Universidad San Sebastián, Santiago, Chile

**Keywords:** *Acidithiobacillia*, *Acidithiobacillus*, mobile genetic element, MGE, plasmid, defense system, restriction-modification system, CRISPR-Cas system

## Abstract

Plasmids are major drivers of microbial evolution, enabling horizontal gene transfer (HGT) and facilitating adaptation through the dissemination of relevant functional genes and traits. However, little is known about plasmid diversity and function in extremophiles. ‘*Fervidacidithiobacillus caldus*’, a meso-thermo-acidophilic sulfur oxidizer, is a key player in sulfur cycling in natural and industrially engineered acidic environments. Here, we present a bioinformatic analysis of the plasmidome, and associated anti-mobile genetic element (anti-MGE) defense systems (defensome), across genomes of this species and metagenomes from diverse natural and industrial settings harboring ‘*F. caldus*’. We identified >30 distinct plasmids, representing five consistent replication-mobilization families. Plasmids ranged in size between 2.5–65 kb, with gene content and plasmid modularity scaling with element size and copy numbers inversely correlating with size. Plasmids carried variable numbers of hypothetical proteins and transposases, with annotated cargo genes reflecting functional differentiation by habitat. Defensome profiling revealed over 50 anti-MGE systems in sequenced ‘*F. caldus*’ isolates, including diverse restriction-modification systems, CRISPR-Cas types IV-A and V-F, and widespread abortive infection and composite defense systems such as Wadjet, Gabija, and Zorya. In environmental populations, an inverse relationship was observed between defensome complexity and plasmidome abundance and diversity, underscoring a pivotal role of the host defensome in modulating persistence, compatibility, and overall plasmid diversity across ‘*F. caldus*’ populations. Yet, other plasmids appeared decoupled from both host abundance and defensome complexity, suggesting potential host shifts, environmental persistence, or differential replication under suboptimal growth conditions for the host. Altogether, these findings point to a modular, functionally diverse adaptive plasmidome shaped by environmental pressures, by the interplay with the host’s defensome, and likely also by other eco-evolutionary processes at play in natural environments. While these associations are compelling, causal relationships remain to be experimentally validated. These insights broaden our understanding of mobile genetic elements in extreme environments and provide a foundation for plasmid-based vector design and synthetic biology applications in acidophiles, with direct implications to biomining and environmental remediation.

## Introduction

Plasmids are major drivers of horizontal gene transfer (HGT) and dissemination of ecologically relevant genes and adaptive traits, i.e., traits currently conferring fitness advantages. Thereby plasmids foster adaptation in its process-based sense, as the evolutionary response of populations to environmental pressures through selection on beneficial traits (Xue et al., 2024; Heuer and Smalla, 2012). However, the composition and distribution of plasmid-borne traits may also be influenced by other evolutionary forces, such as genetic drift or population bottlenecks, particularly in spatially structured or low-density environments (Hülter et al., 2020; Slater et al., 2008). These non-selective processes can affect plasmid persistence, host range, and the fixation of neutral or mildly deleterious elements in a population. HGT mediated by plasmids is a major driver of bacterial diversification, enabling the rapid acquisition of adaptive traits, such as antibiotic resistance, which spread rapidly under anthropogenic pressures (González-Plaza et al., 2019). Plasmids also promote adaptation by increasing gene dosage, enhancing the potential for mutation and diversification of their cargo, and giving rise to phenomena such as heteroplasmy (Ilhan et al., 2019), heterozygosity (Rodriguez-Beltran et al., 2018), and genetic dominance (Rodríguez-Beltrán et al., 2020). These processes can guide the expansion of populations under changing environmental conditions and accelerate bacterial evolution (Rodríguez-Beltrán et al., 2021; Frost et al., 2005).

The plasmid repertoire of prokaryotes is vast and highly diverse, both in terms of adaptive cargo and core backbone genes (e.g., replication and mobilization modules), which vary across hosts and environments (Norman et al., 2009). Substantial variability has been documented in plasmid genome size, architecture, and topology (Shintani et al., 2015), as well as in copy number and host range, mirroring the diversity of their prokaryotic hosts. Plasmids are ubiquitous in nature, being found in both conventional environments such as soil and water, and in extreme habitats such as geothermal sites and hypersaline ecosystems (Perez et al., 2020; Xiang et al., 2015). However, despite their ubiquity, most plasmid research has centered on model microorganisms of medical or industrial relevance, leaving plasmids in extremophiles comparatively understudied. This gap stems in part from the challenges of cultivating and genetically manipulating extremophiles, whose specialized growth requirements and slow replication rates limit experimental tractability and hinder large-scale functional analyses. Yet, studying plasmids in these organisms remains critical to uncover unique adaptive traits and molecular strategies underlying extremophile adaptation and resilience, while also providing genetic parts and design principles valuable for synthetic biology.

Among extremophiles, members of the class *Acidithiobacillia* (Williams and Kelly, 2013) are obligate chemolithoautotrophs and extreme acidophiles that thrive in highly acidic, sulfur- and metal-rich environments. ‘*Fervidacidithiobacillus caldus*’ (provisional genus designation proposed by Moya-Beltrán et al., 2021a; formerly *Acidithiobacillus caldus*), a well-studied member of the *Acidithiobacillia* class, grows optimally at 40–45°C and pH ~ 2.5 (Hallberg and Lindström, 1994). Strains of this species have been isolated from diverse geographical locations and habitats (Nuñez et al., 2014), reflecting its broad adaptability to various abiotic and biotic conditions. Genomic surveys of ‘*F. caldus*’ have revealed moderate genetic diversity among isolates (Nuñez et al., 2014, 2017; Moya-Beltrán et al., 2021a), largely attributed to horizontal gene transfer of mobile genetic elements, including plasmids (Acuña et al., 2013; You et al., 2011; Rawlings, 2005). Yet, the adaptive value of plasmid-encoded functions has not been tested experimentally, and little is known about their distribution, maintenance, or potential fitness costs at the populational level (Baltrus, 2013). Such costs could be particularly detrimental for extremophiles, which typically display starvation-survival strategies, including slow growth, low cell densities, reduced biomass yields, and high degrees of spatial isolation (Coombs, 2009).

In addition to this, an increasing suite of defense systems acting on plasmids has been described in recent years in different model microorganisms (Pradhan et al., 2025; Mamontov et al., 2022; Moya-Beltrán et al., 2021b; Pinilla-Redondo et al., 2020; Marraffini and Sontheimer, 2008), including systems that inhibit plasmid replication or transmission, and limit HGT of their cargo genes (Wheatley and MacLean, 2021; Botelho, 2023). Thereafter, the persistence of plasmids in host cell populations is shaped by a series of interacting factors that sustain the plasmid life cycle (Wein and Dagan, 2020), including plasmid-encoded traits (e.g., replicon type), host factors (e.g., Restriction-Modification systems, Dimitriu et al., 2024), and incompatibilities with co-occurring MGEs (Bouet et al., 2007), all further influenced by environmental selection acting on the accessory genes carried by the plasmid.

Despite renewed interest in plasmid ecology and evolution (fueled by the expansion of metagenomic datasets) their contribution to the structuring of microbial communities and to HGT *in situ* is still poorly understood, particularly in acidic environments. This study investigated the repertoire of plasmids (plasmidome) and defense systems (defensome) of ‘*F. caldus*’ sequenced strains and populations in both natural and industrial acidic environments. By analyzing the occurrence, diversity, and distribution of plasmids, the nature of their backbone gene modules and cargo genes, and associated defense systems, we aimed at elucidating how plasmids contribute to the adaptation of ‘*F. caldus*’ to its environment, and how defense systems influence plasmid persistence and gene flow. Understanding these dynamics is crucial for advancing knowledge of extremophilic biology and could inform biotechnological applications involving bioleaching and bioremediation processes.

## Materials and methods

### Strain isolation and growth conditions

‘*Fervidacidithiobacillus caldus*’ isolates were obtained from acidic hydrothermal and riverine samples collected in February 2018 from multiple sites along the Copahue-Caviahue Volcanic Complex (CVCC) system, located in the Southern Volcanic Region of the Andes. Isolation was performed via direct plating and liquid culture enrichment. For direct isolation, 100 μL of water samples were spread onto solid Mineral Salt Medium (MSM) with trace elements (Dopson and Lindstrom, 1999) supplemented with either elemental sulfur (5 g/L) or tetrathionate (5 mM K_2_O_6_S_4_) as energy sources, and incubated aerobically at 30°C for 15 days. For enrichment, 10 mL of water samples was inoculated into 100 mL of MSM liquid medium with matching energy sources and trace elements, adjusted to pH 2.5 and room temperature, and incubated at 150 rpm. Once visible turbidity developed, cultures were serially diluted and plated under the same conditions. Colonies with distinct morphologies were selected and purified by repeated streaking on solid MSM. Purified strains were routinely maintained in liquid MSM medium with trace elements and a suitable energy source, incubated aerobically at 30°C and 150 rpm, and transferred every 4 weeks. For biomass production cells were grown following recommended optima for ‘*F. caldus*’ (Hallberg and Lindström, 1994). Stationary phase cultures used for nucleic acid purification were processed as in (Moya-Beltrán et al., 2021a).

### Sample collection and field procedures

Sampling was conducted at Cascada de la Culebra (CC), an acidic waterfall located in the Rio Agrio Superior (RAS) at 1,690 m.a.s.l., within the CCVC system early in March 2023. Water samples were collected from the midpoint of the water column in the waterfall plunge-pool, at a low-flow area of RAS-CC as in (Cuevas et al., 2024; Supplementary Table S1). Water was pre-filtered through 8 μm Whatman grade 2 cellulose filters disks (particle-associated community) and collected on 0.22 μm MCE membrane disk filters (Millipore) for bulk water sample (25 L) metagenomic sequencing using a 500 mL Nalgene serial vacuum filtration system. Filters were stored at −20°C in the field and thereafter at −80°C until DNA extraction and sequencing.

### Genomes, MAGs and metagenomes

Genome and metagenome sequencing, as well as assembly, have been described previously (Moya-Beltrán et al., 2021a; Degli Esposti et al., 2023). Public genomes (*n* = 17) and Metagenome Assembled Genomes (MAGs) of ‘*F. caldus*’ (*n* = 9) and reference strains (*Thermithiobacillus tepidarius* DSM 3134 T), were obtained from the public WGS NCBI Genome database in October 2024. When available sequence read archives (SRA) for the genomes were also downloaded. For taxonomically targeted recovery of public metagenomes containing *Acidithiobacillia* class members and ‘*F. caldus*’ representatives we used Sandpiper v0.3.0 (Woodcroft et al., 2024) and downloaded the cognate files from NCBI via the SRA Run accession numbers. Quantification of *Acidithiobacillia* and ‘*F. caldus*’ in each environmental sample was done via phylogenomic inference as in (Degli Esposti et al., 2023) using the bac120 housekeeping genes matrix (Parks et al., 2020), applying identity thresholds of 85% for class-level and 95% for species-level detection, with stringent criteria of e-value < 1e-5 and query coverage 90%.

Metagenomes contributed by this study were deposited at the National Center for Biotechnology Information (NCBI) under the BioProject accession ID PRJNA914835. The complete list of genomes and metagenomes used in this study, their corresponding statistics, along with relevant metadata can be found in Supplementary Table S1.

### Plasmid contigs identification

Genomic contigs from sequenced strains and MAGs were analyzed to identify candidate plasmid sequences based on gene content and structural features. Open reading frames (ORFs) were predicted and functionally annotated using the SqueezeMeta v1.6.3 pipeline (Bankevich et al., 2012; Tamames and Puente-Sánchez, 2019) and databases GenBank, eggNOG, KEGG, and Pfam (Clark et al., 2016; Huerta-Cepas et al., 2017; Kanehisa, 2002; Finn et al., 2014), updated in October 2023. Annotations were cross-validated with RAST (Rapid Annotation using Subsystem Technology; Aziz et al., 2008). Orthologous protein clusters, defined here as Protein families (PF), were defined using ProteinOrtho v6.3.1 (Lechner et al., 2011) with a 60% identity and 60% coverage threshold based on bidirectional BLASTp best hits; all else as described in (Moya-Beltrán et al., 2021a). Additional similarity searches were performed using BLAST and PSI-BLAST algorithms (Altschul et al., 1997). Mapping and coverage validation were conducted with Bowtie v1.2.2, and samtools v1.1 and default parameters defined previously (Degli Esposti et al., 2023). Candidate plasmid contigs were identified based on the presence of plasmid hallmark genes, including replication initiator proteins (Rep), mobilization modules (Mob), and stabilization systems. Candidate target proteins were confirmed by annotation and domain architecture against the CDD database v.3.16 using CDsearch (Marchler-Bauer et al., 2002), RPS-BLAST v2.2.26 and hhsearch (Fidler et al., 2016), using default parameter values. For robust classification of conjugative modules, MOBscan, CONJscan and MOBFinder, were applied following the recommended workflows, thresholds, and classification schemes detailed in the original publications (Garcillán-Barcia et al., 2020; Feng et al., 2024; Cury et al., 2020).

### Anti-MGE defense systems prediction

General prediction and classification of defense system types in ‘*F. caldus*’ genomes, MAGs, and selected metagenomes was done using DefenseFinder 11 v1.0.8 (Tesson et al., 2022) and PADLOC v1.0.0 (Payne et al., 2022), applying default parameters. Individual predictions were merged via protein ID, and congruence was checked manually. Further refinement of the predictions was done using dedicated tools. CRISPR/Cas systems were reassessed by using CRISPRFinder (Grissa et al., 2007), and Cas-associated proteins were classified according to established frameworks (Moya-Beltrán et al., 2021b; Makarova et al., 2020; Makarova and Koonin, 2015). Restriction-Modification (R-M) systems were validated through sequence similarity, conserved domains identification, and domain architecture analyses against REBASE (Roberts et al., 2023). Type-II R-M systems were also predicted by using the rmsFinder pipeline (Shaw et al., 2023). Type-II methyltransferases target sequences in plasmids and cognate chromosomes of the taxon were predicted by similarity searches using the target recognition domain (TRD) sourced from REBASE 409 (accessed in October 2024). All defense-related proteins were clustered by genomic vicinity as in (Moya-Beltrán et al., 2021b), and manually curated to refine the defense system assignments.

### Phylogenomic and phylogenetic analyses

A cladogram was constructed on the basis of the phylogenomic analysis performed using a set of 120 bacterial single-copy marker proteins (bac120; Parks et al., 2020) derived from ‘*F. caldus*’ isolate genomes, MAGs, and reference strains (*T. tepidarius* DSM 3134ᵀ genome as outgroup). Protein alignments were generated using MUSCLE with default parameters in MEGA X suite v10.2.3 (Kumar et al., 2018). Maximum-likelihood (ML) phylogenetic trees were inferred in MEGA X using the Le_Gascuel_2008 substitution model. Initial trees for heuristic searches were generated using the Neighbor-Joining and BioNJ algorithms, based on a pairwise distance matrix estimated with the JTT model, and the topology with the best log-likelihood score was selected. Rate heterogeneity among sites was modeled with a discrete Gamma distribution (5 categories, +G, shape parameter = 0.7819), and a proportion of sites was allowed to be invariable (+I, 29.09%). The final dataset included 28 sequences and 9,923 aligned amino acid positions. The best-fitting evolutionary model was selected based on the Bayesian Information Criterion (BIC) using the MEGA model selection tool. Dendrograms used only for referential representations of sequence similarity (e.g., replicases and relaxases), were generated using the Neighbor-Joining method implemented in MEGA X, based on multiple sequence alignments produced with MAFFT v7.520 with the L-INS-i strategy (parameters: maxiterate 1000 and localpair) to optimize alignment accuracy. Plasmidial heme-copper oxidases (HCO) A1-2 CyoB protein sequences, along with ‘*F. caldus*’ cognate chromosomal HCO-A1-1a and HCO-A1-1b CyoB variants and reference CyoB proteins of *Acidithiobacillus ferrooxidans* ATCC 23270 TY (type HCO-A1-1a) and *T. tepidarius* DSM 3134 TY (type HCO-A2-1) were used to construct CyoB phylogenetic trees. HCO classification scheme follows Moya-Beltrán et al., 2021a. Multiple sequence alignments were performed using MAFFT v7.520 as above. Phylogenetic inference was carried out using FastTree v2.1.11 with the Whelan and Goldman (WAG) substitution model and CAT approximation (20 rate categories). The resulting CyoB ML tree was rooted with the *T. tepidarius* HCO-A2-1 sequence and depicted as a cladogram. Cladograms and phylograms were visualized by using the ggtree v3.16.0 R package. The CyoB supporting alignment (doi: 10.6084/m9.figshare.29546420) and the original, unaltered phylogram, preserving full evolutionary distances, and branch length information is available in Newick format via FigShare (doi: 10.6084/m9.figshare.29546288).

### Statistical analysis and data visualization

Data analysis and visualization were conducted using the R language (R version 4.4.3) with ggplot2 v3.5.1, tidyverse v2.0.0, patchwork v1.3.0, maps v3.4.3, ggrepel v0.9.6, and viridis v0.6.5 packages. Statistical significance was assessed using the Wilcoxon signed-rank test using the ggsignif v0.6.4 R package. Genetic context and clustering visualizations were conducted using Clinker v0.0.20 program (Cameron et al., 2021). Commands and scripts used in this study are publicly available at the following DOIs: genomes, MAGs and metagenomes (doi: 10.6084/m9.figshare.29481266.v3), plasmid contigs identification (doi: 10.6084/m9.figshare.29481323.v4), anti-MGE defense systems prediction (doi: 10.6084/m9.figshare.29481206.v2), and phylogenomics and gene neighborhood analyses (doi: 10.6084/m9.figshare.29429270.v4).

## Results and discussion

### Genomic profiling of plasmids in ‘*Fervidacidithiobacillus caldus*’

**Figure 1:**
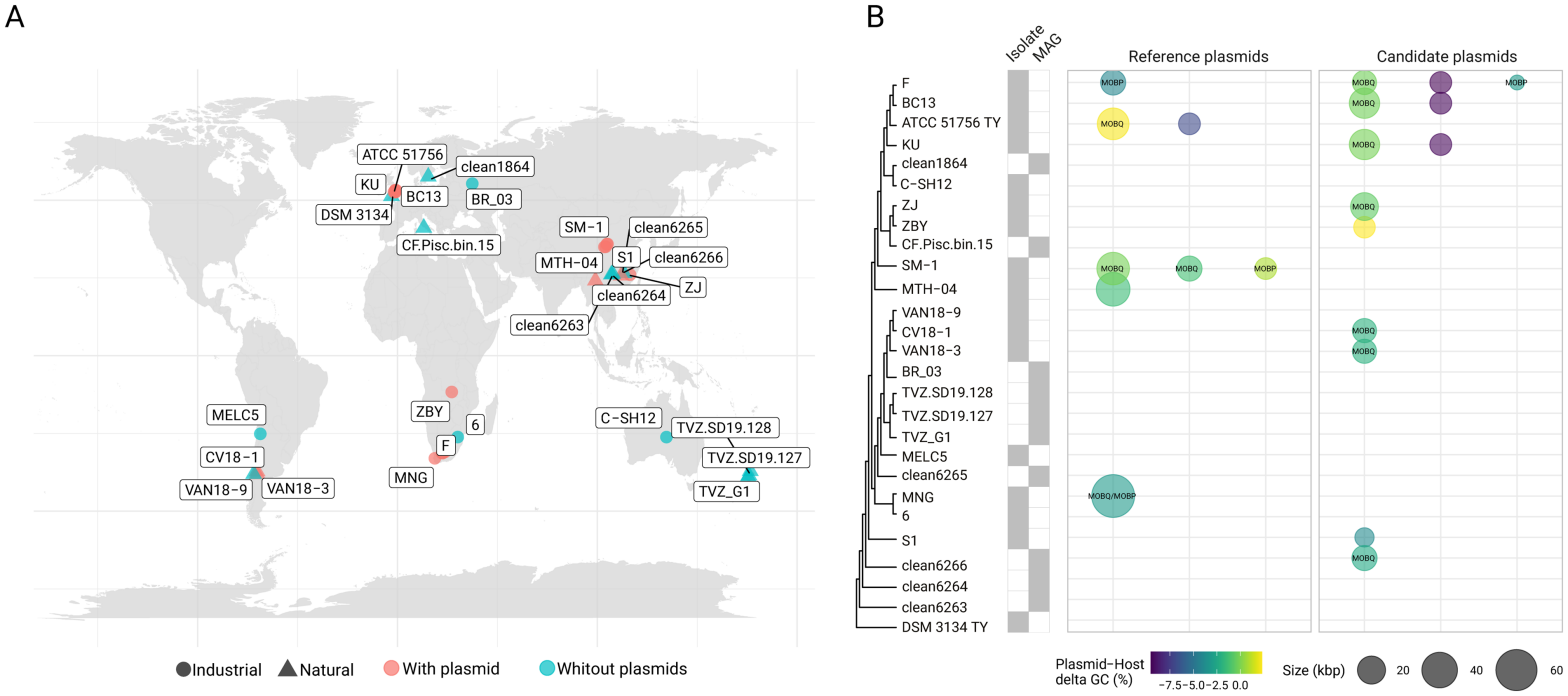
Prevalence and phylogenetic distribution of plasmids in ‘*F. caldus*’ genomes. **(A)** Geographical origin and source (natural or industrial) of ‘*F. caldus*’ isolates and MAGs included in this study. The map was generated using the maps v3.4.3 package in R. Strains harboring plasmids are shown in light red, while plasmid-free strains are shown in light blue. **(B)** Cladogram derived from a Maximum-likelihood (ML) phylogenetic tree based on the concatenated alignment of 120 bacterial single-copy marker proteins (*bac120*, Parks et al., 2020) using 17 isolate genomes and 9 MAGs available in GenBank as of July 2024. Sequences were aligned using MUSCLE (default parameters), yielding a final alignment of 9,923 amino acid positions (ungapped). For tree inference, the JTT substitution model and a discrete Gamma distribution of rate heterogeneity (+G) with 100 ultrafast bootstrap replicates were used, as implemented in the MEGA X suite v10.2.3 (Kumar et al., 2018). The resulting tree is rooted using *Thermithiobacillus tepidarius* DSM3134 T as outgroup, consistent with its placement as a sister taxon to *Acidithiobacilliaceae* (Moya-Beltrán et al., 2021a). Plasmid occurrence is represented by a dot: dot size reflects known or inferred plasmid size, and color indicates the difference in G + C content between the plasmid and the host chromosome. For mobilizable plasmids, the type of relaxase (as classified by Garcillán-Barcia et al., 2020) is indicated inside the dots.

**Figure 2:**
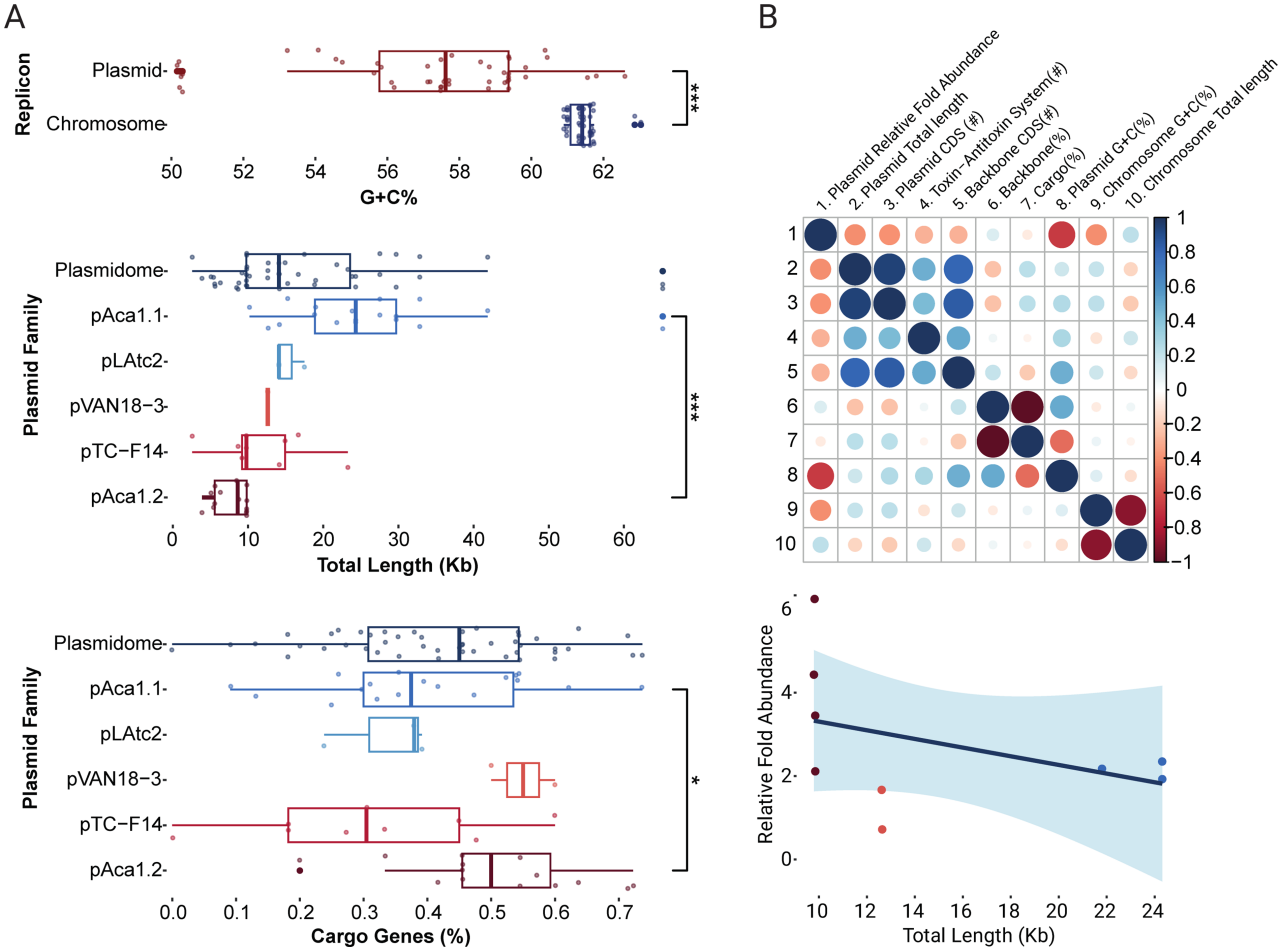
Distribution of genomic features across *‘F. caldus’* plasmids. **(A)** Boxplot of the distribution of plasmid G + C content, size (kb), and proportion of cargo genes. Plasmids GC content (%) is significantly lower than their cognate chromosomes (*p* < 0.001) and vary widely in size (2.6–65 kb; median: 14 kb). Significant differences were also observed in plasmid size and cargo gene content across plasmid families (*p* < 0.001 and *p* < 0.05, respectively). Significance was assessed using the Wilcoxon signed-rank test implemented via the ggsignif package in R. **(B)** Correlation analyses between plasmid genomic metrics. The correlogram (top) shows Pearson correlation coefficients between selected features, with blue indicating positive correlations and red negative. Color intensity reflects correlation strength. The scatterplot (bottom) illustrates the inverse relationship between plasmid size and inferred plasmid copy number (trend line and confidence interval), based on normalized read depth with respect to the chromosome, and indicated here as relative fold abundance.

Novel plasmids, including variants of previously described ‘*F. caldus*’ plasmids, were identified in the majority of globally sampled strains (Supplementary Table S1a), indicating >75% plasmid prevalence across the species (Figures 1A,B). A similarly high prevalence of plasmids (in the range of 70–85%) has been reported in other bacteria (e.g., Mora-Hernández et al., 2021), yet according to current understanding is higher than in other *Acidithiobacillia* class species (Beard et al., 2021). The identified replicons varied in size, ranging from 2.6 to 65 Kb, with a median size of 14 Kb (Figure 2A) and had a relative abundance with respect to the cognate chromosomal replicons of 0.6 to 6.2 fold (Supplementary Table S2). The calculated plasmids copy numbers correlated negatively with the size of the elements (Figure 2B). This suggests that the candidate secondary replicons of the *Acidithiobacillia* would all be plasmids with a medium to low copy number, as is indeed the case for experimentally evaluated plasmids of the class (Rawlings and Tietze, 2001). With few exceptions, the % G + C content of all these plasmids was lower than that of the respective host genomes (Supplementary Table S2).

The *in silico* analysis of publicly available ‘*F. caldus*’ genomes (17 derived from isolates and 9 corresponding to MAGs; Supplementary Table S1a) revealed that the number of plasmids varied from one to three plasmids per strain, with 35.7% of the strains harboring one plasmid, 71.4% multiple plasmids, and 17.6% being devoid of plasmids. Even if lower than the plasmid carriage (8–10 plasmids per strain) observed in clinical and environmental bacteria (Ombui et al., 2000; Neyaz et al., 2020; Walter et al., 2020), including certain acidophiles (e.g., 8 plasmids in *Acidiphilium* spp., Li et al., 2020), the co-carriage of multiple plasmids per isolate is of significance particularly in the light of the limited evidence for plasmid incompatibility in ‘*F. caldus*’ (Gardner et al., 2001) or in acidophiles in general (Beard et al., 2021).

### Backbone functional modules underpin ‘*Fervidacidithiobacillus caldus*’ plasmid families and coexistences

#### Replication modules

**Figure 3:**
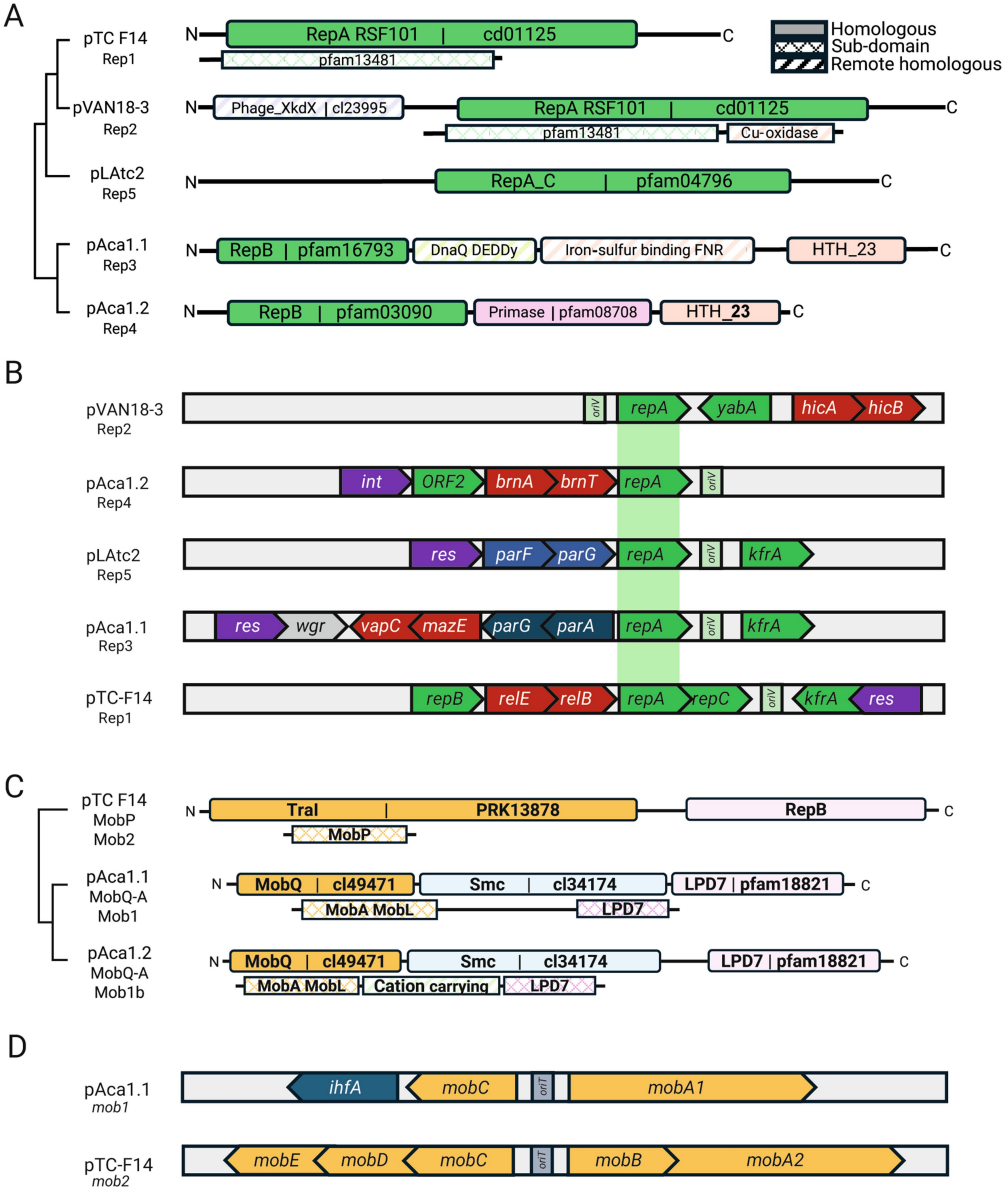
Backbone module organization of ‘*F. caldus*’ plasmids. **(A)** Replication modules RepA_1 to RepA_5 identified across ‘*F. caldus*’ plasmids, differing in replicase type (single- or multi-domain) and associated protein domain architecture. **(B)** Gene vicinity of RepA per replicon type, showing adjacency of replication (green), stabilization (red) and partition genes (blue) and the replication origin location (*ori*V region). **(C)** Clustering by mobilization module type (*mob1* vs. *mob2*) and protein domain architecture of identified relaxases (MOBQ and MOBP_A families) in ‘*F. caldus*’ plasmids. **(D)** Gene architecture of mobilization modules *mob1* and *mob2*, highlighting differences in relaxase family, accessory proteins (RAPs), and *ori*T region location. Gene architectures support the classification of plasmids into functional families and offer insights into compatibility and inheritance mechanisms.

To determine the basis of their coexistence and obtain hints on the fitness costs of the plasmidome, we classified ‘*F. caldus*’ plasmids into distinct families based on their core gene content (Table 1). Given that plasmid incompatibility (defined as the inability of two co-resident plasmids to be stably inherited by daughter cells in the absence of selection relies on features of the replicon, such as the origin of replication and/or the partitioning system; Novick, 1987), we first analyzed these aspects across the recovered plasmids. Sequence-based clustering of plasmid-encoded proteins revealed 351 clusters, with 40% corresponding to conserved backbone protein families (PFs) and 60% representing accessory PFs, several of which conformed distinct gene modules (Figure 3). Sequence analysis identified five distinct replication modules, differing in replicase type (single or multidomain, Figure 3A) and replicon organization (Figure 3B). All modules harbored a variant of the replicative helicase (Supplementary Table S2). The *rep* gene from plasmids represented by pTC-F14 (RepA_1, 291 aa) and pVAN18-3.1 (RepA_2, 435 aa) encoded distinct proteins with sequence similarity to the replicative helicase of the RSF1010 plasmid family (pfam13481, Scherzinger et al., 1997). The pTC-F14 replicase (RepA_1; IncQ-2b) shares with other IncQ-family replicons the presence of three replication related genes, *repB*, *repA* and *repC*, encoding the primase, the helicase and the iteron-binding protein, respectively (Dorrington and Rawlings, 1990; Rohrer and Rawlings, 1992), in the proximity of a 22-bp iteron based *ori*V (Rawlings and Tietze, 2001). pTC-F14-like plasmids were detected in strains MNG, SM-1 and S1 (pTcM1, pLAtc1, pS1, pCanu) with conserved organization and *ori*V, supporting a rolling-circle replication mechanism (Loftie-Eaton and Rawlings, 2012) with medium copy numbers (12–16 copies per chromosome, Rawlings and Tietze, 2001). In contrast, pVAN18-3.1 (RepA_2) lacked homologous primase and iteron-binding proteins but encoded a large multidomain replicase (> 400 aa) and a small (132–135 aa) DNA binding protein, suggesting functional divergence. The latter showed similarity to ORF2 (NF038291) from the replication region of plasmid pAB02 and related *Acinetobacter* plasmids, supporting a functionally divergent replication strategy for these medium sized plasmids. The replicase from pAca1.1-like (RepA_3, 437 aa) and pAca1.2-like (RepA_4, 335 aa) plasmids encoded a three-domain protein containing a replicase, a primase and a C-terminal DNA-binding domain and were found adjacent to a predicted *ori*V and to a replication associated CDS encoding either KfrA or an ortholog of ORF2 (pAB02_ORF2). The fifth type of replication module uncovered in ‘*F. caldus*’ plasmid pLAtc2 (RepA_5, 329 aa) encoded a RepA_C (pfam04796) that is only 28.6% similar to its best matching hit in ‘*F. caldus*’ (RepA, pTC-F14). This replicase was encoded next to the *ori*V and convergent to a KfrA-encoding gene, a plasmid-specific nucleic acids binding protein (NAP) found also in other plasmids of the *Acidithiobacillia* class (Beard et al., 2023), and recently shown, in the conjugative plasmid RA3 of *Pseudomonas aeruginosa*, to mediate low-copy-number plasmid segregation during cell division through interactions with the segregosome proteins IncC (ParA) and KorB (ParB (Lewicka et al., 2021). These rep-modules distributed differentially among strains of the species (Supplementary Table S3), with several evident plasmids concurrencies or exclusions, underscoring compatibility between replicons Rep1: Rep3, Rep1: Rep5 and Rep3: Rep4. Rare concurrence Rep3: Rep5 and Rep2: all (Supplementary Figure S1a) suggests, yet does not confirm, a degree of functional incompatibility. Based on the occurrence of a single replicon-type per strain, all these replicons are inferred to be self-incompatible, except for Rep4 found in variant plasmids related to pAca1.2 in several strains (Supplementary Figure S1b). Also, one plasmid carrying two replicons could be identified as a cointegrate of a pTC-F14-like plasmid (RepA_1) and a pAca1.1-like (RepA_3), with evidence of compromised integrity of the *repABC* replicon (pTcM1 in strain MNG, van Zyl et al., 2008).

**Table 1:** Plasmid families identified in ‘*F. caldus*’ sequenced strains and MAGs.

Plasmid family	Representative plasmid	Size range (Kb)	Relaxase MOB type	1ry RepA type	2ry RepA type	PAR system	TA systems (#)	Total genes (#)	Cargo genes (#)	Cargo genes w/ function (#)	Backbone (%)	Concurrence per strain
1 (*n* = 5)	pTC-F14	9.8–14,2	PA	Rep1	–	–	1	21	10	4	52,4	3, 4, 5
1, 3 (*n* = 1)	pMNG_TcM1	65,2	Qa, PA	Rep3	Rep1	Par1	3	103	44	23	35,9	1,3
3 (*n* = 13)	pAca1.1	27.5–32.8	Qa	Rep3	–	Par1	2	39	21	11	46,2	1, 4, 5
5 (*n* = 2)	pLAtc2	14.1	Qa	Rep5	–	Par2	0	20	5	3	75,0	1, 3, 4
2 (*n* = 2)	pVAN183_JAAOMN010000091	12.6	Qa	Rep2	–	–	1	13	7	4	46,2	–
4a (*n* = 5)	pAca1.2	9.8	-	Rep4a	–	–	1	11	6	3	45,5	1, 3, 4, 5
4b (*n* = 4)*	pS1_LZH01000935	5.6–8.5	-	Rep4b	–	–	1	11	7	2	36,4	1, 3, 4a, 4c, 4d, 5
4c (*n* = 2)	pZJ_LZYG01000035	5.1–8.8	-	Rep4c	–	–	2	12	5	3	58,3	3, 4b, 4d
4d (*n* = 2)	pZBY_LZYF01000110	3.9–5.4	-	Rep4d	–	–	1	7	4	3	42,9	3, 4b, 4c

*n*: number of strains/genomes/MAGs carrying the plasmid; (*): integrated in a chromosomal contig.

#### Partitioning modules

Partitioning systems were consistently located adjacent to replication genes, forming seamlessly integrated modules (Figure 3B). Sequence analysis identified ParFG-*parH*-like systems in ‘*F. caldus*’ plasmids (McLeod et al., 2017), resembling those of the multiresistance plasmid TP288 (Baxter and Funnell, 2014), and comprising four ParF (COG1192; pfam13614) and three ParG variants. ParF proteins shared 37.7–83.8% identity among ‘*F. caldus*’ plasmids and 41.9–52.6% with ParF from TP288, while ParG orthologs were more divergent (23.5–32.2%), suggesting adaptive differentiation of centromere-binding factors. A conserved 4-nt tandem repeat (5′-CTAT-3′), flanked by AT-rich sequences, was also identified near the *parFG* loci and likely represents a centromere-like site in pAca1.1-like plasmids. The Par system components were consistently associated with the pAca1.1 plasmid family, the most widespread in ‘*F. caldus*’ (12 strains and 2 MAGs), supporting the notion that partition is the most important determinant of plasmid stability (Baxter and Funnell, 2014). However, their presence alone does not guarantee compatibility, as co-occurrence of plasmids with different Par variants was observed in one case [strain F: pAca1.1-like (JAAOML010000407) pTC-F14 (JAAOML010000340)]. Since partition systems are ubiquitous in low-copy number plasmids (or generally absent in high-copy number plasmids), we infer that all pAca1.1_like plasmids are low copy number plasmids. This aspect is supported by plasmid copy number inference (1.67 plasmids/per chromosome; Supplementary Table S2) and experimentally proven to be low (1 copy of pAca1.1 every 2 chromosomal copies) in the ‘*F. caldus*’ ATCC 51756^T^ (Haristoy, 2012).

#### Mobilization modules

To improve the classification of plasmid families in ‘*F. caldus*’, we characterized their gene modules responsible for dispersal and stabilization within hosts. A total of 13 unique relaxases were identified, belonging to MOBQ or MOBP_A families (Garcillán-Barcia et al., 2009). As shown in Figure 3C, MOBQ relaxases occurred as two variants distinguished by their conserved motif organization. MOBP_A relaxases were less frequently detected. Relaxases clustered into two mobilization module types, *mob1* and *mob2* (Figure 3D). The *mob1* modules (8 variants), encoded a MobA relaxase of the MOBQa type, along with three small relaxase-accessory proteins (RAPs). These RAPs include a MobC ortholog, an IHF histone-like protein, and a DNA-binding protein (HTH_23: PF13384; DUF742: PF05331) of unclear function. Presence of IHF coding genes and IHF-binding sites in the *ori*V region of *Acidithiobacillia* class plasmids has been previously reported (Beard et al., 2023; Chakravarty et al., 1995; Moya-Beltrán et al., 2023) and inferred to have a role in DNA-bending linked to plasmid replication and/or transfer facilitation (Di Laurenzio et al., 1995; Biek and Cohen, 1989). In turn, *mob2* modules (5 variants) encoded a MobA relaxase of the MOBP_A type and four conserved accessory proteins (MobBCDE). The *mob1* module was found in all pAca1.1-related plasmids (*n* = 11, Rep3-type) and pLAtc2-like plasmids (*n* = 2, Rep5-type), which are all medium to large plasmids, while the *mob2* module was invariably linked to smaller-sized IncQ2 plasmids (*n* = 5, Rep1-type), including well-characterized pTC-F14 (van Zyl et al., 2003). In addition to the relaxase domain (pfam03432) of the MOBP_A family, the MobA_mob2 proteins contained a second conserved domain present in RepB primases (pfam16793) and clustered with 4 defined, yet variable, Mob accessory proteins (sequence-level similarity range: 21.2–77.8%). Despite this variability, their conserved occurrence and organization reinforce previous findings on the essential role of *mobA, mobB*, and *mobC* RAPs in pTC-F14 plasmid mobilization in ‘*F. caldus*’ (van Zyl et al., 2003). The MobA-RepB fusion proteins displayed a high degree of conservation, with 71.3–100% sequence similarity among ‘*F. caldus*’ plasmids and 73.0–89.2% similarity to its only known homolog in the *Acidithiobacillia* class, the pTF-FC2 plasmid. Probable origins of transfer *ori*T could be predicted in all plasmids, at the intergenic region between the *mobA* gene and divergently transcribed RAPs (Supplementary Figure S2). The sequence of the *ori*T was highly conserved between plasmids with the same *mob*-type. Still all *ori*Ts conformed to a general palindromic configuration described previously for plasmids pTF1 and pTC-F14, being adjacent to a highly conserved predicted nick site. All ‘*F. caldus*’ plasmids lacked conjugation genes, yet the widespread occurrence of mobilization genes indicate they necessarily co-opt the type IV secretion machinery of other self-transmissible MGEs (Smillie et al., 2010; Meyer, 2009).

#### Stabilization modules

Twenty nine out of thirty plasmids in the ‘*F. caldus*’ plasmidome encoded at least one toxin-antitoxin (TA) system, with 46.7% carrying multiple systems (Supplementary Table S2). These were invariantly type II TA systems, which are frequently associated with MGEs and HGT in other organisms (Fraikin et al., 2020). Five toxin families were identified: RelE, VapC, HicB, BrnT, and Doc, each with 1–4 variants. RelE PF was the most widespread, with multiple subtypes (4 RelE, 1 HigB, 1 YafQ). In agreement with general knowledge (Deter et al., 2017), TA operons were typically bicistronic, consisting of toxin-antitoxin gene pairs, (e.g., *relBE, brnTA*, etc.); an exception was pLAtc1, which harbored non-cognate *relE* and *vbhA* genes in separate loci. TA systems were frequently located between plasmid backbone genes related to replication (*repA, kfrA*), mobilization (*mobA, mobE*), or partitioning (*parG*), reinforcing their role in plasmid stabilization. Two organizational patterns were observed: canonical (*antitoxin-toxin*, e.g., *vapBC, relBE, dinJ-yagQ, hicBA, phd-doc*) and non-canonical (*toxin-antitoxin*, e.g., *brnT-brnA, higB-higA*) (Heaton et al., 2012). Interestingly, the non-canonical configuration was found exclusively in smaller plasmids (e.g., pAca1.2-like plasmids carrying *brnT-brnA*), possibly reflecting an aspect of the regulation of compact replicons. However, specific studies investigating the effects of reversed gene orientation on the maintenance efficiency of small versus larger plasmids are unavailable. All TA toxins in ‘*F. caldus*’ targeted translational processes (Harms et al., 2018); they are predicted to function as ribonucleases (*RelE* targets mRNA, *HicA* degrades mRNA independently, *VapC* cleaves initiator *tRNAfMet*), translation elongation inhibitors (*Doc* phosphorylates EF-Tu, disrupting translation), or act on undefined RNA targets (*BrnT*). Larger plasmids frequently combined 2 or 3 TA systems with different specificities [*pAca1.1*: *vapBC* (tRNA) and *relBE* (mRNA)]. Also, plasmids coexisting in the same strain tended to encode distinct TA types [*pAca1.1 (vapBC, relBE) vs. pAca1.2 (brnTA)*], or variants within the same type *[pF_JAAOML010000123 pAca1.1-like (relE1) vs. pF_JAAOML010000310 pTC-F14-like (relE2)]*, suggesting independent plasmid stability control. This strategy likely enhances plasmid persistence while preventing exclusion of competing plasmids with similar backbone structures (Cooper and Heinemann, 2000).

### Organizational principles of the ‘*Fervidacidithiobacillus caldus*’ plasmidome

*‘F. caldus*’ plasmids segregated into two main size classes with distinct organizational features (Table 1; Supplementary Table S4). Small plasmids (<10 kb), exclusively harbored Rep4-type replicons and non-canonical TA systems, lacked mobilization and partition modules, and were mostly cryptic, with few or no functionally annotated genes. Despite their minimal gene content, they were widespread, detected in >50% of strains and often coexisted as multiple variants in certain strains (e.g., ZJ/ZBY). Some appeared integrated into the chromosome or larger MGEs (e.g., var4b), consistent with the presence of FimB-FimE-Int type integrases in some pAca1.2-like plasmids; however, this interpretation remains tentative and warrants experimental confirmation. Coverage analysis further indicated these small plasmids were present at significantly higher copy numbers (3-6x chromosome levels) than coexisting larger plasmids, suggesting dosage-dependent roles. Although functionally uncharacterized, their persistence and widespread distribution across strains indicates they are probably more than neutral genomic passengers. In contrast, ‘*F. caldus*’ larger plasmids (>10 kb) exhibited greater functional complexity, incorporating partitioning, mobilization, and stabilization modules, supporting long-term maintenance and potential horizontal dissemination within the species and broader microbial communities at lower copy numbers (<2 copies per chromosome). Mobilization was inferred in all large plasmids, primarily via MOBQa-type relaxases, with differences in both replicon architecture (at least 4 different replicon types) and cargo load, which accounted for 25 to 50% of their total sequence length.

Absence of the gene operons encoding the conjugative bridge in these plasmids hints on their dependency on chromosomally encoded or co-resident systems encoded in Integrative Conjugative Elements or ICE (Acuña et al., 2013; Moya-Beltrán et al., 2023; Flores-Ríos et al., 2019) for complete mobilization. In larger plasmids, stabilization systems - mostly type II TA systems, scaled with plasmid size, with approximately one TA system per 10 kb, indicating a role in maintenance under low-copy-number conditions. Collectively, insights gained on the natural architecture of native ‘*F. caldus*’ plasmids reveal rules for compatibility and maintenance and offer practical guidance for synthetic vector design. For instance, in high-load plasmids, inclusion of a ParFG partitioning system and size-adjusted TA modules is likely essential to ensure stability. In contrast, small to mid-sized vectors intended for transient use may not require such systems.

### Core and accessory components of the ‘*Fervidacidithiobacillus caldus*’ defensome

**Figure 4:**
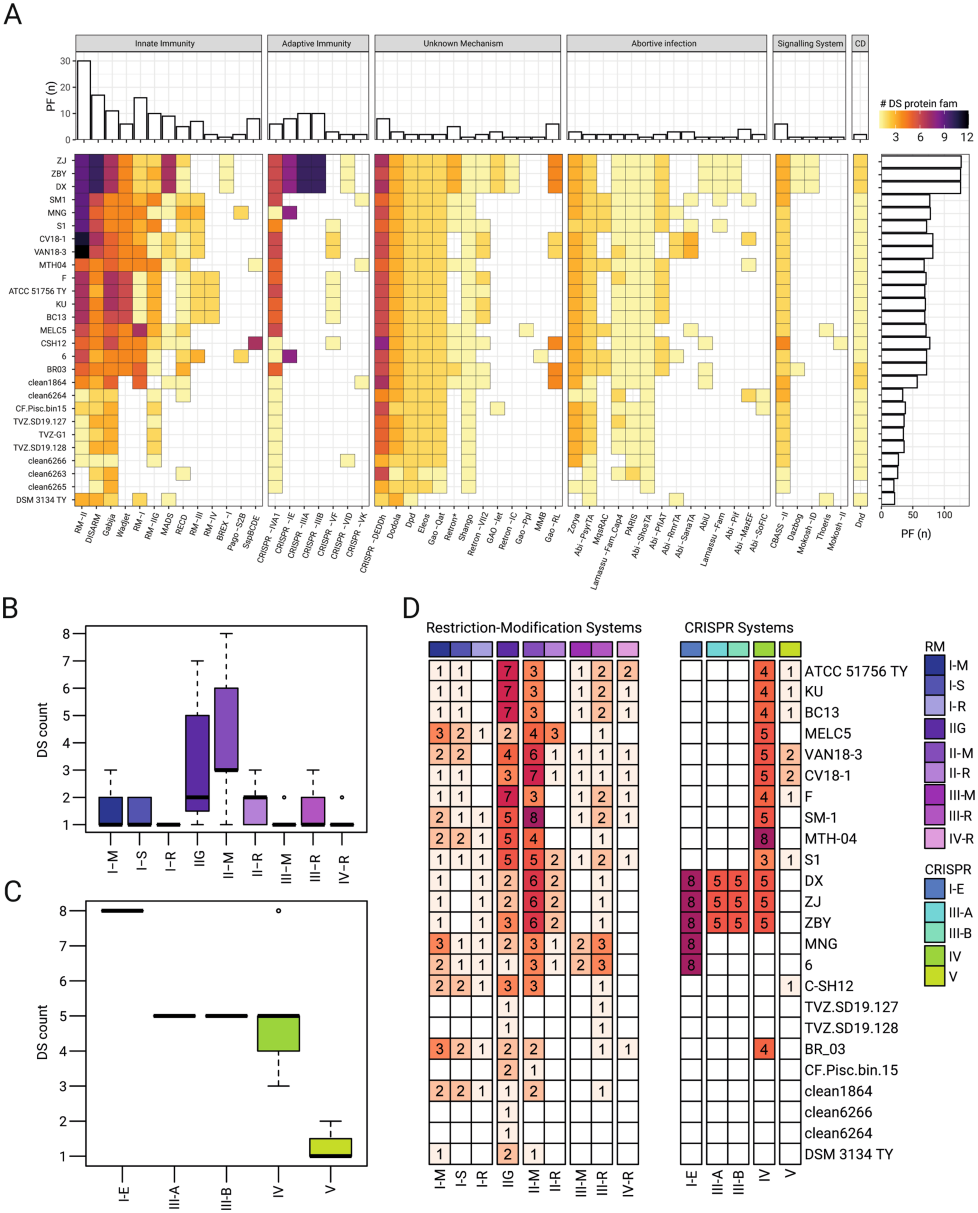
Diversity and distribution of defense systems in ‘*F. caldus*’ genomes and MAGs. **(A)** Heatmap showing the presence/absence of 50 predicted defense system (DS) types across genomes and MAGs of ‘*F. caldus*’. DSs were classified and categorized after (Tesson et al., 2022; Payne et al., 2022; Millman et al., 2022), and colored by the number of protein families (PF) identified per DS in the corresponding genomes. Core DSs (present in ≥90% of genomes/MAGs), sporadic (4–17 strains), and rare (<3 strains) distributions are highlighted. **(B,C)** Boxplot showing the number of predicted restriction-modification (R-M) systems per genome, grouped by R-M subtype (I–IV, IIG) or CRISPR-Cas system subtype (III, IV and V) across genomes and MAGs. **(D)** Heatmap displaying the distribution per strain of RM systems and CRISPR systems PFs clustered by orthology using the ProteinOrtho orthology detection tool v6.3.1 (Lechner et al., 2011) at 60% identity and 60% coverage thresholds.

A total of 54 distinct DS-types were identified across ‘*F. caldus*’ genomes and MAGs (Figure 4A; Supplementary Figure S3; Supplementary Table S5), spanning well-characterized functional categories of defense (Bernheim and Sorek, 2020; Millman et al., 2022), as well as non-canonical (Doron et al., 2018) and putatively novel systems (Tesson et al., 2022; Payne et al., 2022). While many of these systems are classically linked to antiviral defense, several are also known to restrict MGEs, including plasmids (Pradhan et al., 2025; Roisné-Hamelin et al., 2024; Deep et al., 2022; Pinilla-Redondo et al., 2020; Marraffini and Sontheimer, 2008). Our results confirm the presence and diversity of these systems in ‘*F. caldus*’, particularly restriction modification (RM)-systems (Figures 4B,C) and CRISPR-Cas systems (Figures 4B,D), reinforcing their potential role in shaping plasmid persistence and mobility within this species.

The abundance and distribution of DSs varied widely across strains and was generally lower in MAGs. A core set of 19 DS-types, spanning 32 genes across 16 gene neighborhoods, were present in at least 90% of genomes and MAGs (Figure 4A). In contrast, 21 DS subtypes were sporadically distributed across ‘*F. caldus*’ strains (detected in 4 to 17 strains), while 10 were rare (present in fewer than 3 strains), with distribution patterns broadly correlating with the strains’ geographical origin or environmental source (Supplementary Table S1b). These patterns suggest both vertical conservation and horizontal acquisition across environmental contexts. Core DSs included diverse gene clusters targeting nucleic acid degradation [e.g., Wadjet (Deep et al., 2022), BREX (Goldfarb et al., 2015), DISARM (Aparicio-Maldonado et al., 2021)], synthesis inhibition (e.g., CBASS, Duncan-Lowey and Kranzusch, 2022), and phosphorothioation (e.g., Dnd, Jiang et al., 2023). Several abortive infection systems (e.g., PARIS, PsyrTA, ShosTA) and novel or composite systems (e.g., Gabija, Shango, and Zorya) were also prevalent. Of note were the type IV CRISPR-Cas and the type-I Wadjet systems, both implicated in anti-MGE interference, and detected in all strains of the species, yet absent from available MAGs. Recent investigations demonstrate that plasmid-borne type IV-A CRISPR-Cas modules can hijack host adaptation machinery to acquire spacers *in trans* and transcriptionally silence replication or conjugation genes of rival plasmids, thereby curbing their horizontal spread (Benz et al., 2024). Meanwhile, the SMC-family Wadjet complex detects the topology of closed-circular DNA and, through loop-extrusion-coupled cleavage, selectively eliminates plasmids smaller than ~100 kb (Pradhan et al., 2025). In complete genomes of ‘*F. caldus*’ these systems locate within, or between, know ICE (Acuña et al., 2013) or within predicted ICE-like elements (data not shown), further supporting their role in the resolution of inter-MGE conflicts and/or MGE proliferation (Moya-Beltrán et al., 2021b). Rare or strain-specific DSs included several location-restricted systems such as (i) a DISARM-RM hybrid cluster present in the megaplasmids of SM-1 and MNTH-04, (ii) prophage-linked RM clusters associated with the AcaML1 prophage (Covarrubias et al., 2018); (iii) Type I-E CRISPR-Cas systems restricted to industrial strains from China and Africa (Zhang et al., 2016); and (iv) several integron-encoded systems (e.g., Gao-related). Less frequent DSs like BREX-I, Mokosh-II, Thoeris, among others, were detected in isolated genomes (Figure 4).

The most frequent DS type and most abundant DS proteins were those linked to DNA restriction-modification (R-M). A total of 350 putative RM-proteins were identified across ‘*F. caldus*’ genomes and MAGs, clustering into 35 distinct gene cluster arrangements. Except for two MAGs lacking RM genes, all genomes encoded 2 to 26 RM-systems, averaging 19 per genome. MAGs generally harbored fewer RM loci. No RM cluster was shared across all genomes, although the type IIG system was the most widespread, found in >85% of strains and 7 of 8 MAGs. Type II RM systems were the most abundant, comprising 19 clusters with substantial sequence diversity, occurring mostly in individual strains (Figures 4C,D).

Besides RM systems, CRISPR-Cas systems with known or potential relevance in plasmid interference were detected in the genomes analyzed. These included class systems of subtype IV-A, and class 2 systems of subtype V-F. Type IV was the most widespread, found in >85% of sequenced strains (and in 1 MAG), and in agreement with previously reported occurrence in the species and the *Acidithiobacillia* class (Moya-Beltrán et al., 2021b) it is hypothesized to contribute to MGEs conflict resolution via interference between plasmids and integrative conjugative elements (Benz et al., 2024). Interestingly, over 50% of the strains carried one or two Class 2 CRISPR-Cas type V-F (Cas12f) effectors, i.e., small (400–700 amino acids) RNA-guided endonucleases distantly related to transposon-encoded TnpB nucleases (Altae-Tran et al., 2021) that target DNA in a 5′ T-rich PAM-dependent manner, producing staggered double-stranded breaks (Wu et al., 2021), and whose collateral ssDNA cleavage capacity has been leveraged in diagnostics and gene editing (Chen et al., 2018; Bigelyte et al., 2021). Beyond these roles, experimental studies with protein variants from extreme acidophiles (e.g., *Sulfoacidibacillus thermotolerans*, ex. *Acidibacillus sulfuroxidans*, AsCas12f1) have confirmed Cas12f-mediated plasmid interference in *E. coli* (Wu et al., 2021), suggesting that other orthologs of these compact effectors may also be capable of adaptive immune functions against plasmids complementing or substituting for other anti-MGE systems within the species’ broader defensome.

### Linking plasmid diversity and defense complexity in environmental populations of ‘*Fervidacidithiobacillus caldus*’

**Figure 5:**
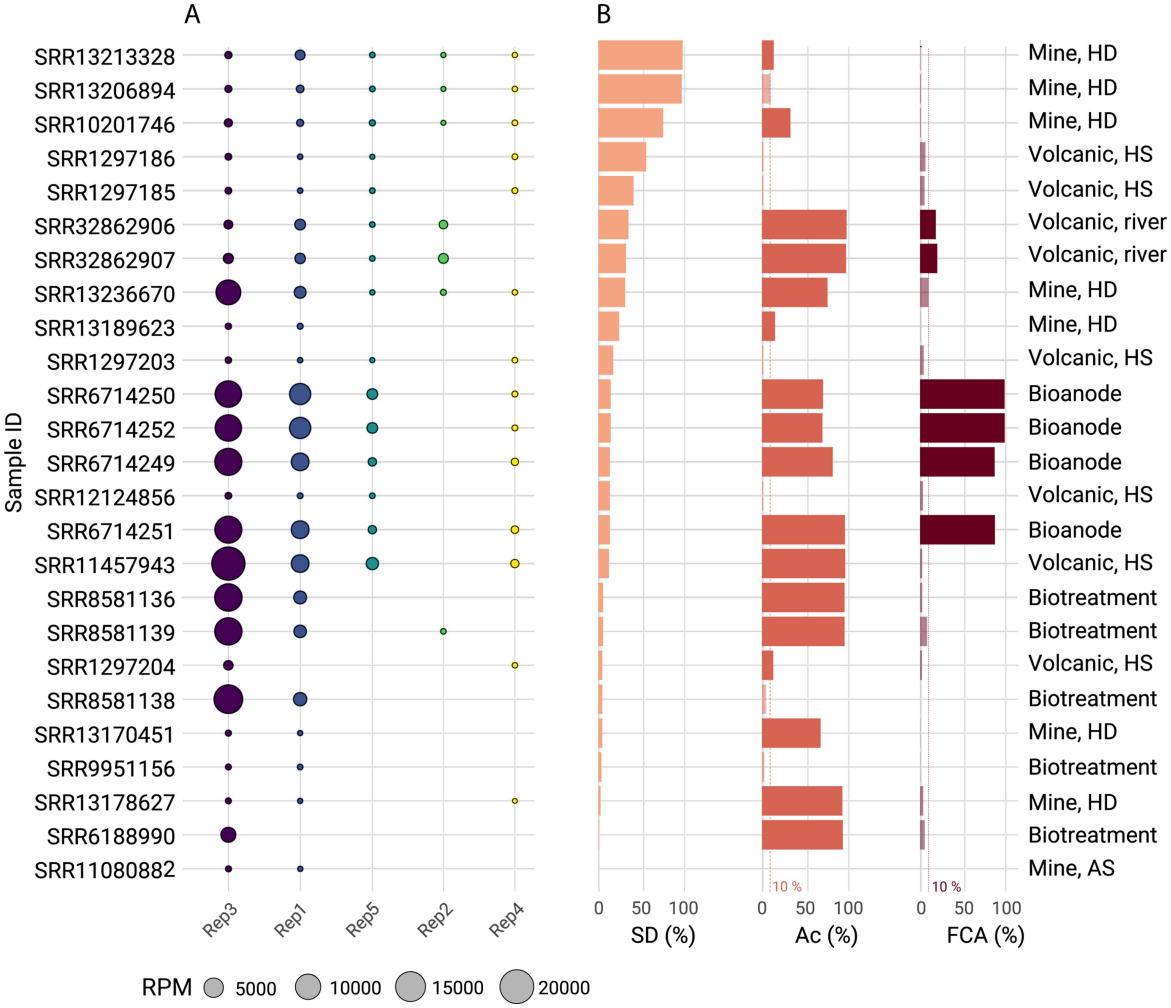
Global distribution of ‘*F. caldus*’ plasmid families and replicon types across acidic environments. **(A)** Occurrence and relative abundance in reads per million (RPM) of the five ‘*F. caldus*’ plasmid families (Rep1–Rep5) across 25 metagenomic samples with presence of *Acidithiobacillia* or ‘*F. caldus*’ at an abundance threshold >10%, selected using the Sandpiper platform. Metagenomes were sourced from diverse acidic ecosystems, including geothermal springs, volcanic river systems, acid mine drainages, and engineered bioleaching systems (Supplementary Table S1b). Plasmids proteins were clustered at 70% identity using CD-HIT and mapped to the metagenomes using BLAST. Replicon type was inferred by mapping against the curated plasmid backbone the protein dataset derived from sequenced strains and MAGs. The abundances of *Acidithiobacillus*, ‘*F. caldus*’, and REP 1–5 proteins were calculated from read counts and normalized to relative abundances (100%). The pAca1.1-like family (Rep3) and pTC-F14-like family (Rep1) were the most widespread, consistent with their prevalence in isolate genomes. **(B)** Percentual abundance of ‘*F. caldus*’ defense systems in the retained metagenomes (DS %), along with the relative abundance of the class (Ac %) and the ‘*F. caldus*’ species (FCA %). The defense systems of ‘*F. caldus*’ were evaluated by performing a BLASTp of the defensome proteins (retrieved from *Acidithiobacillus* genomes) against the metagenomic assemblies, applying a 90% identity threshold to retain high-confidence matches.

Among 25 *Acidithiobacillia*-containing metagenomes, only 7 surpassed the abundance threshold set for ‘*F. caldus*’ (>10%; Supplementary Table S1b). This subset included metagenomes from geographically and environmentally diverse acidic ecosystems, including geothermal springs, volcanic river systems, acid mine drainages, and engineered biotechnological systems. Presence and relative abundance of Rep1–Rep5 replicon marker proteins across the assembled datasets (Figure 5A) was consistent with genome-based results generated in this study, with the pAca1.1 plasmid family being the most prevalent across samples with presence of ‘*F. caldus*’ (Rep3, >75%) followed closely by the pTC-F14 family (Rep1, >60%). The metagenomic contigs recovered showed evidence of conserved backbone structures for the five plasmid families, with variations mostly confined to the plasmid cargo genes or accessory gene pool (Supplementary Table S6). One variant plasmid of the pTC-F14 family, displaying only 64% average similarity to the *mob2* module of the reference pTC-F14, was identified in the acidic riverine system of RAS-CC, expanding the repertoire of P-type mobilizable plasmids in the taxon by contributing novel gene content and organizational diversity. Also, comparative analyses of reference plasmids, and plasmid-borne contigs in draft genomes and metagenomes, provided clear evidence for the origin of larger plasmids (~65 kb, pTcM1) described previously in strains MNG or CSH12 (Haristoy, 2012), hinting to cointegration events of pAca1.1 and pTc-F14 family plasmids. Stability of such large plasmids in ‘*F. caldus*’ strains could be compromised, as no evidence of pCSH12 was detected in the strain (provided by Dr. D. E. Rawlings) following multiple serial passages under laboratory conditions.

Plasmids from different families followed independent occurrence and abundance patterns, with all plasmid families excepting pVAN18-3 (Rep2) correlating positively (R > 0.67) with the total abundance of the host in the sample and negatively (*R* < -0.32) with the total abundance of ‘*F. caldus*’ defense systems in the sample (Figure 5B; Supplementary Table S7). These associations suggest that, for most plasmids, the complexity of the host defensome may influence their presence and/or persistence within the host population, ultimately shaping the overall structure of the plasmidome. In contrast, the atypical distribution of pVAN18-3 points to a more complex interplay between host abundance, defensome architecture, and eco-evolutionary processes such as selection, drift, and dispersal, highlighting the multifactorial nature of plasmidome dynamics, beyond deterministic selective pressures alone.

### Functional traits encoded by plasmid cargo across genomes and metagenomes

**Figure 6:**
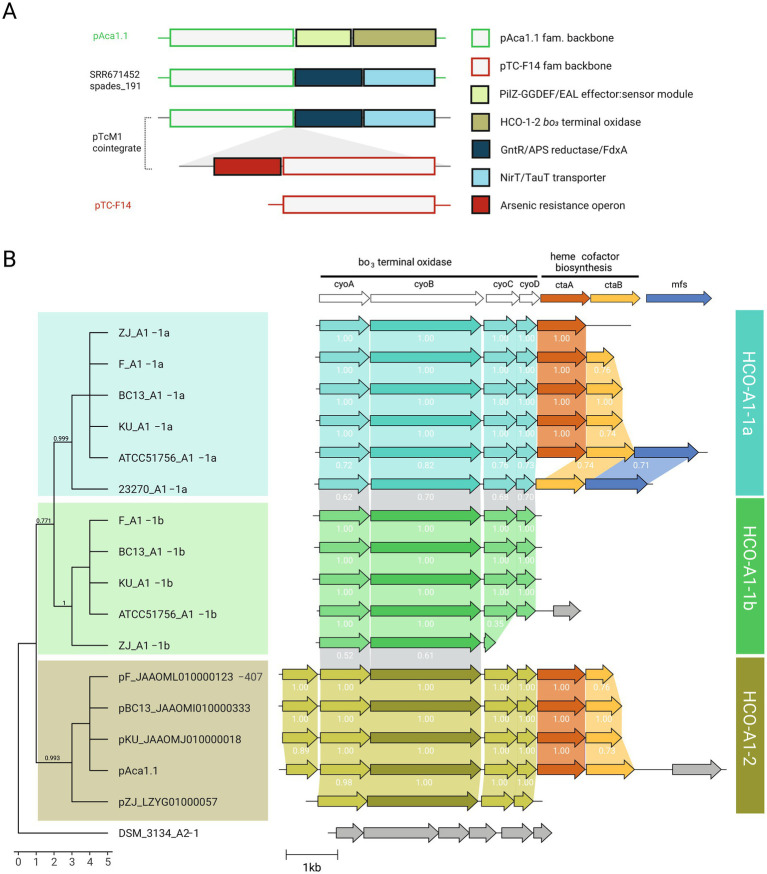
Adaptive cargo of ‘*F. caldus*’ plasmids. **(A)** Schematic representation showing the proposed trajectories of several cargo modules along ‘*F. caldus*’ plasmids. Reference plasmid pAca1.1 and pTC-F14 backbone regions as well as adaptative cargo modules PilZ-GGDEF/EAL, HCO-A1-2 cytochrome *bo_3_* terminal oxidase, ferredoxin/APS reductase/GntR-family transcriptional regulator, ABC-type NirT/TauT sulfonate transporter and arsenic resistance operon along plasmids pAca1.1, pTcM1 and pTC-F14, and the SRR6714252_spades_191 metagenome recovered plasmid-like contig are shown. **(B)** Comparative analysis of HCO-A1 type cytochrome *bo_3_* terminal oxidase loci in ‘*F. caldus*’ plasmids and their corresponding host strains. The cytochrome *bo_3_* terminal oxidase and linked heme cofactor biosynthesis gene neighborhoods from A1-1a And A1-1b chromosomal subunit II *cyoB* variants, along with the plasmidial A1-2 *cyoB* variants are mapped to a CyoB protein sequence-based tree. The tree was inferred using the Wheland-and-Goldman (WAG) substitution model as implemented in FastTree v2.2, based on a multiple sequence alignment of 755 amino acid positions (716 positions after gap removal) generated using MAFFT L-INS-i. The ML-tree was rooted using the *Thermithiobacillus tepidarius* DSM 3134 T A2-1 CyoB variant, in accordance with previous phylogenomic studies that place this lineage as basal within the *Acidithiobacillia* class (Moya-Beltrán et al., 2021a). Bootstrap values are shown at nodes. Branch lengths on the x-axis have been proportionally scaled to improve the visual alignment of gene neighborhood diagrams displayed alongside the tree. The unmodified phylogram with original branch lengths is available in Newick format via FigShare (doi: 10.6084/m9.figshare.29546288). Genetic context visualizations were constructed using Clinker.js tool (Cameron et al., 2021). Names and color keys for CyoB variants are according to (Moya-Beltrán et al., 2021a).

To gain further insight into these interplay, we analyzed the cargo gene complement from both genome and metagenome derived plasmid contigs (Figure 6A). This set entailed a total of 248 non redundant proteins, 59 of which had robust functional assignments (Supplementary Table S8). An additional 38 predicted proteins could be assigned to transposases (ISL3, IS4, IS5, IS30, IS66, ISChy9), and 151 PFs remained as hypotheticals. Twelve PFs with predicted function were found in both genomes and metagenomes from industrial habitats, linked to plasmid families pAca1.1, pTC-F14, pLAtc2 and pAca1.2 in decreasing frequency. In turn, 15 PFs linked to signaling, regulation and transport of nutrients, metals, and metalloids, or to competitive interactions, were only found in metagenomes (Supplementary Table S8). Among PFs exclusively associated to genomes of the species (*n* = 32), those encoding a variant heme copper oxygen oxidase of the A1 subtype-2 (HCO-A1-2) described previously in the species (Moya-Beltrán et al., 2021a), were the most frequent (Figure 6B). These were in all cases encoded in pAca1.1 family plasmids, supporting a role for this plasmid type in adaptation to varying redox conditions or oxygen levels, and in the evolution of HCO oxidases in the class. In contrast, pAca1.1 family plasmids from natural populations - recovered from hot spring metagenomes and engineered biotechnological systems - carried distinct cargo genes, including two operons predicted to participate in the uptake and assimilation of organosulfonate compounds as alternative sulfur sources (Kertesz, 2000). In marine ecosystems, sulfonates serve as key intermediates in trophic exchanges between phytoplankton and heterotrophic bacteria (Durham et al., 2019), raising the possibility that analogous mechanisms may facilitate community-level nutrient exchange in acidic environments and/or involving *Acidithiobacillia* spp. Evident presence of pAca1.1 (and pTC-F14) family plasmids in samples lacking detectable ‘*F. caldus*’ (e.g., sludge metagenome, Figure 5) further suggests that these elements may have dispersed to other members of the *Acidithiobacillia* class, reinforcing the ecological relevance of plasmid-mediated horizontal gene transfer in shaping microbial interactions and adaptability in acidic environments.

Other top-ranking COG functional categories among cargo proteins included regulation and signal transduction (Supplementary Figure S4). The presence of multiple GGDEF/EAL domain proteins harboring both diguanylate cyclase (GGDEF) and phosphodiesterase (EAL) domains essential for the synthesis and degradation of the second messenger c-di-GMP, alongside PilZ-type effectors (Castro et al., 2015), and key transcriptional regulators such as the flagellar master regulator FlhC (Wang et al., 2006), the multidrug efflux regulator MarR (Grove, 2013), and the arsenic resistance regulator ArsR (Busenlehner et al., 2003), points to sophisticated plasmid-encoded signaling networks involved in motility, biofilm formation, and metal/metalloid resistance. These traits have been linked previously to plasmids in bacteria (e.g., Choi et al., 2015) and in ‘*F. caldus*’ (van Zyl et al., 2008; Moya-Beltrán et al., 2023). The documented presence of plasmid-borne arsenic resistance cassettes in ‘*F. caldus*’ isolates from arsenopyrite-rich bio-oxidation plants (Kotze et al., 2006), coupled with their exclusive detection in plasmids from strains inhabiting industrial environments in our dataset, highlights the niche-adaptive nature of this cargo in high-arsenic habitats and suggests host–plasmid coevolution driven by localized selective pressures.

Plasmids of the pVAN18-3 family were found in high abundance in environments where ‘*F. caldus*’ was scarce (Figure 5). These plasmids carried genes involved in cyclic-di-GMP signaling, a central regulatory system that controls critical adaptive traits in acidophilic bacteria, including biofilm formation, motility, cell envelope remodeling, and responses to environmental stressors (Moya-Beltrán et al., 2019). In acidic, resource-limited, or fluctuating conditions, such traits are essential for microbial survival and competitiveness. The observed decoupling of plasmid abundance from that of their canonical host may arise from horizontal gene transfer to alternative hosts, environmental persistence as extracellular DNA, or stabilization mechanisms that affect the plasmid-to-host ratio, such as the host entering a dormant state. Among these scenarios, we favor the latter, considering the habitat type (riverine water column) and the environmental conditions at the sampling site (RAS-CC; pH 2.5, 18°C), which are suboptimal for ‘*F. caldus*’ and may promote dormancy or low metabolic activity in the host population. Yet, this remains to be experimentally tested. Altogether, these results suggests that pVAN18-3 plasmids, via their cyclic-di-GMP signaling cargo, may facilitate population- and/or community-level adaptation under suboptimal or fluctuating environmental conditions, and emphasize the role of plasmids as key agents in microbial resilience, horizontal gene flow, and niche expansion within extreme environments.

Conjunctly, several of the plasmid-encoded traits uncovered can be directly or indirectly linked to acid stress. These traits reflect adaptations to physicochemical constraints imposed by low pH, as well as responses to the geochemical pressures commonly associated with acidic environments. This supports the interpretation that acidity acts as a powerful ecological filter shaping the plasmid-borne functional repertoire of ‘*F. caldus*’. While limited metadata precludes a comprehensive environmental comparison, the recurrent presence of these traits in strains from acidic and metal-rich habitats underscores the central role of acidity in driving the evolution, dissemination, and persistence of mobile adaptive elements.

## Conclusion

This study provides a comprehensive analysis of the architecture and diversity of the plasmidome of ‘*F. caldus*’, an extremophilic sulfur-oxidizing bacterium inhabiting highly acidic, metal-rich and moderately hot environments. By integrating genomic and metagenomic datasets from 17 strains and multiple natural and engineered acidic habitats, we identified over 30 native plasmids belonging to 5 distinct families, defined by their unique replicon and mobilization modules. Plasmid families varied in their backbone architecture, including replication, partitioning, and stabilization systems, reflecting selective pressures favoring plasmid maintenance across populations and environmental conditions. Compatibility patterns, co-occurrence profiles, and a documented case of cointegrate formation, provided evidence of plasmid-plasmid interactions and evolutionary dynamics occurring within the species. While core features were consistent, plasmid cargo genes varied markedly between habitat types. Strains from industrial environments shared similar adaptive genes, including arsenic resistance operons and organosulfonate assimilation pathways. In contrast, environmental sequences from geothermal and volcanic sites harbored differentiated cargo, entailing different signal transduction, regulatory and transport mechanisms, relevant in the responses to redox stress or nutrient limitation. These differences point to localized host–plasmid coevolution driven by specific environmental pressures.

Although a rich diversity of plasmid-targeting defense systems was detected across genomes and metagenomes, observed associations between defensome complexity and plasmid carriage was plasmid-dependent, supporting the view that adaptive cargo, not merely backbone architecture or host compatibility, plays a central role in plasmid success across ‘*F. caldus*’ populations. Taken together, insights obtained in this study into plasmid compatibility, persistence, and cargo-mediated adaptation offer a conceptual and practical framework for plasmid engineering, with implications for synthetic biology, bioleaching, and bioremediation applications in extreme environments, and positions ‘*F. caldus*’ as a valuable model for exploring host–plasmid–defensome interactions in extremophilic microbiomes.

## Data Availability

The genome and metagenome sequences analyzed in this study can be found in the online NCBI repository, with the accession numbers listed in Supplementary Table S1. Novel datasets presented in this study have been deposited in GenBank under the BioProject accession number: PRJNA914835.

## Supplementary material

Supplementary Figure S1 Compatibility relationships among ‘*F. caldus*’ plasmids based on replicon co-occurrence. **(A)** Summary matrix displaying the co-occurrences of plasmid replicon types across ‘*F. caldus*’ strains. The number of plasmids per strain is indicated. **(B)** Compatibility relationships among plasmids inferred from patterns of co-occurrence across strains. All replicon types were self-incompatible, except for Rep4-type plasmids, showing up to three variant per strain. Replicon pairs Rep1:Rep3, Rep1:Rep5, and Rep3:Rep4 frequently coexisted, with the latter being the most prevalent combination. Rep3:Rep5 concurrence was infrequent and Rep2 with any other replicon were mutually exclusive, suggesting a potential functional incompatibility. One cointegrate plasmid carrying two replicons (Rep1 and Rep3) was also identified, suggesting recombination between plasmids of different families.

Supplementary Figure S2 Predicted origin of transfer (oriT) of mobilizable plasmids in ‘*F. caldus*’. Predicted oriT regions were consistently located between the mobA relaxase gene and the divergently transcribed RAPs, regardless of the mob module. Sequence conservation was high among plasmids of the same mobilization module type but variable between types. All oriTs conformed to a palindromic structure adjacent to a predicted nick site, resembling oriTs from plasmids pTF1 and pTC-F14 (Rawlings, 2005; van Zyl et al., 2003). In bold conserved bases between plasmids of the same mob type and in red the inferred nick site.

Supplementary Figure S3 Defense systems diversity and distribution in ‘*F. caldus*’ genomes. (A) Frequency histogram showing the number of defense systems (DS) that could be assigned to a single genomic locus, and therefore clustered as “defense islands”. (B) Number of DS-associated unique protein families (PFs), and their prevalence in ‘*F. caldus*’ genomes. The prevalence was defined as the number of genomes in which a given DS PF was identified divided by the total genomes analyzed. (C) Total number of DS-associated PFs identified in each ‘*F. caldus*’ genome sequence, ranked from highest to lowest. PFs were defined by clustering orthologs using ProteinOrtho (Lechner et al., 2011) with thresholds of 60% identity and 60% coverage. Defense systems were classified after (Tesson et al., 2022; Payne et al., 2022; Bernheim and Sorek, 2020; Millman et al., 2022; Doron et al., 2018).

Supplementary Figure S4 Functional assignment of ‘*F. caldus*’ plasmid encoded protein cargo. Protein sequences recovered from non-backbone plasmid regions were used as queries for homology searches using the COG database by using eggNOG. Number of coding sequences assigned to a given COG top level categories.
