# Supplementary figures and images for: Exploring the eco-evolutionary role of plasmids and defense systems in ‘Fervidacidithiobacillus caldus’ extreme acidophile
Source: Front Microbiol. 2025 Aug 11;16:1610279. doi: 10.3389/fmicb.2025.1610279 (PMC12375680; doi:10.3389/fmicb.2025.1610279)

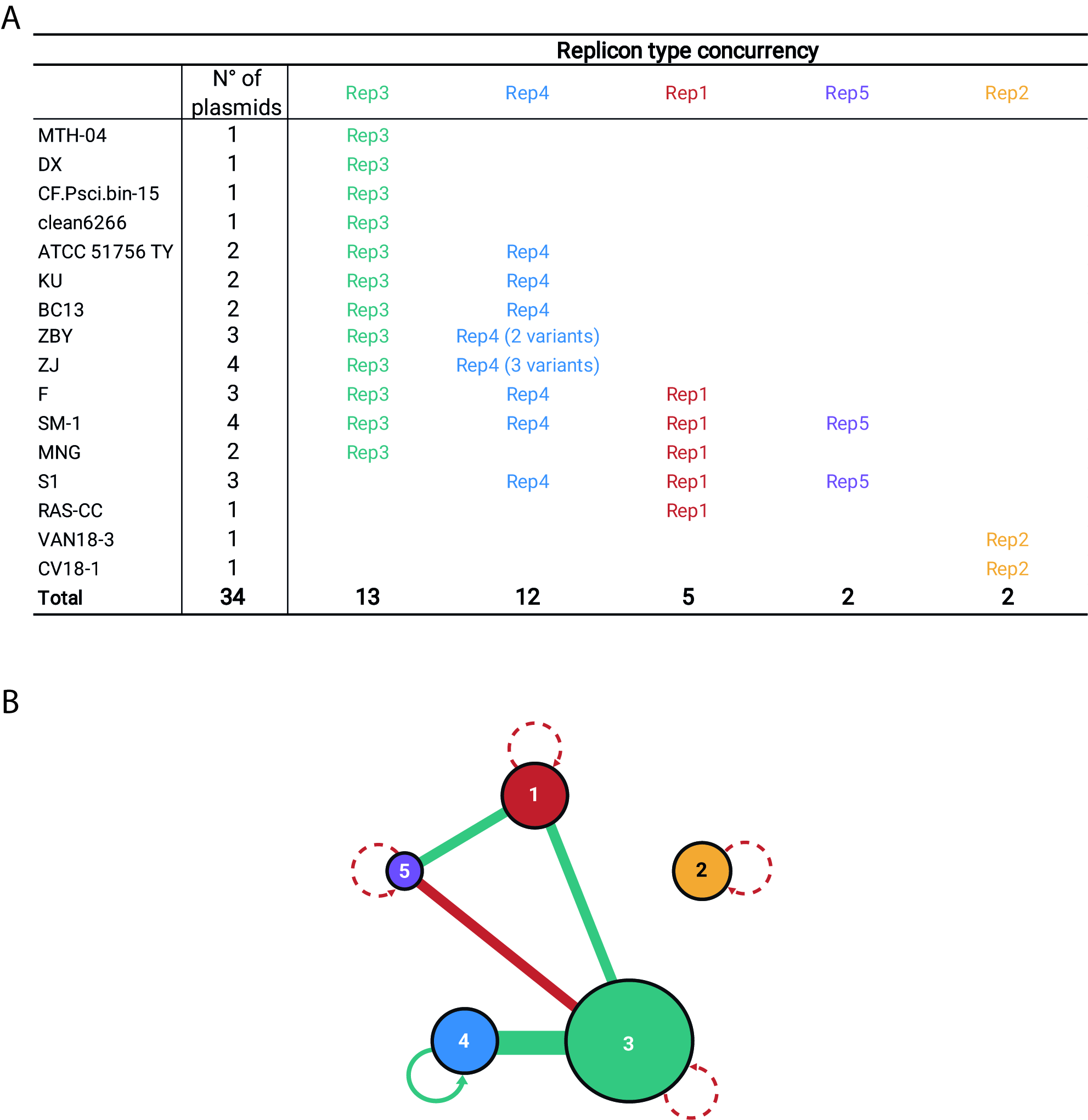

Supplement: Supplementary Figure S1 — Compatibility relationships among ‘F. caldus’ plasmids based on replicon co-occurrence. (A) Summary matrix displaying the co-occurrences of plasmid replicon types across ‘F. caldus’ strains. The number of plasmids per strain is indicated. (B) Compatibility relationships among plasmids inferred from patterns of co-occurrence across strains. All replicon types were self-incompatible, except for Rep4-type plasmids, showing up to three variant per strain. Replicon pairs Rep1:Rep3, Rep1:Rep5, and Rep3:Rep4 frequently coexisted, with the latter being the most prevalent combination. Rep3:Rep5 concurrence was infrequent and Rep2 with any other replicon were mutually exclusive, suggesting a potential functional incompatibility. One cointegrate plasmid carrying two replicons (Rep1 and Rep3) was also identified, suggesting recombination between plasmids of different families. [file Image_1.TIF]

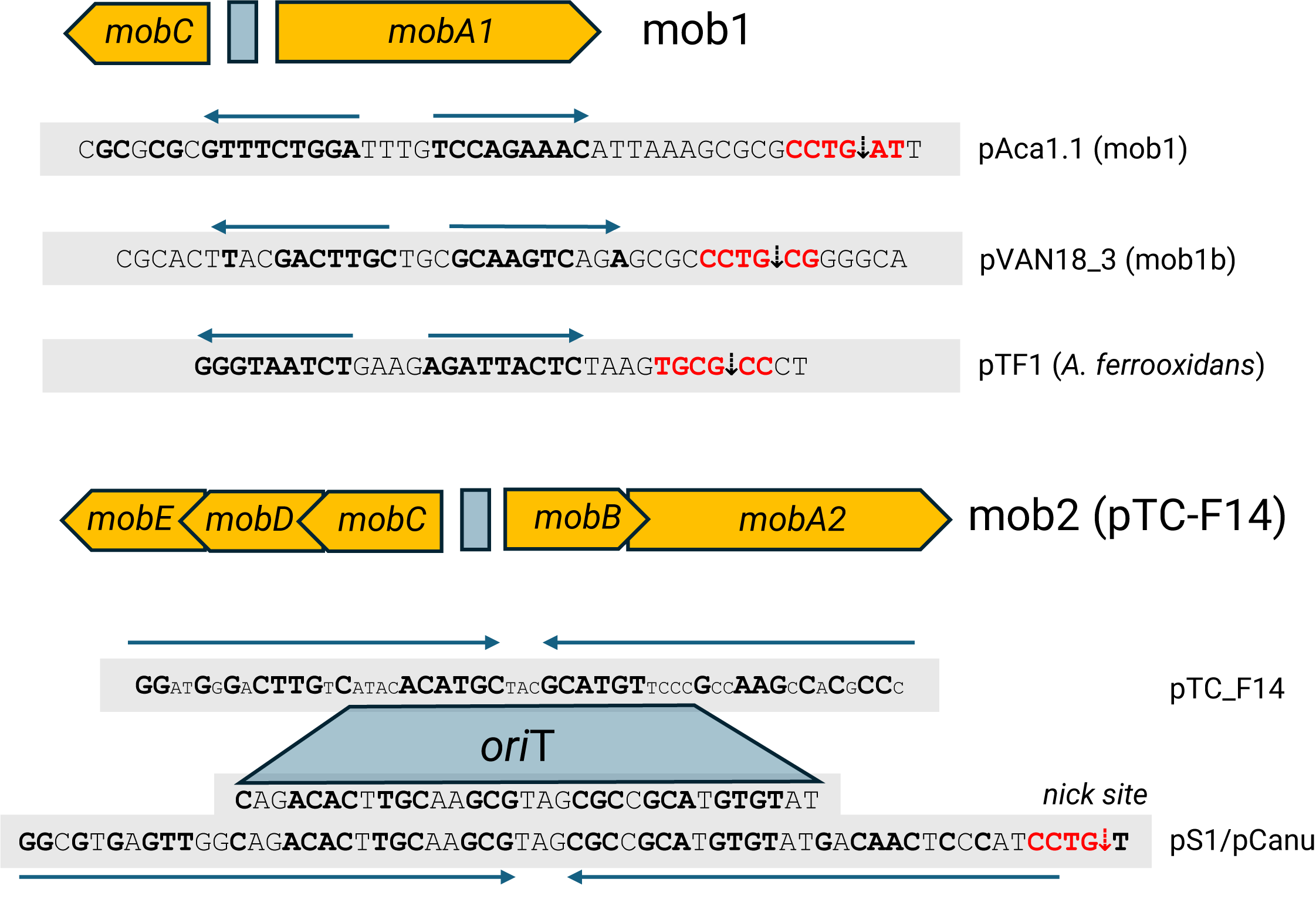

Supplement: Supplementary Figure S2 — Predicted origin of transfer (oriT) of mobilizable plasmids in ‘F. caldus’. Predicted oriT regions were consistently located between the mobA relaxase gene and the divergently transcribed RAPs, regardless of the mob module. Sequence conservation was high among plasmids of the same mobilization module type but variable between types. All oriTs conformed to a palindromic structure adjacent to a predicted nick site, resembling oriTs from plasmids pTF1 and pTC-F14 (Rawlings, 2005; van Zyl et al., 2003). In bold conserved bases between plasmids of the same mob type and in red the inferred nick site. [file Image_2.TIF]

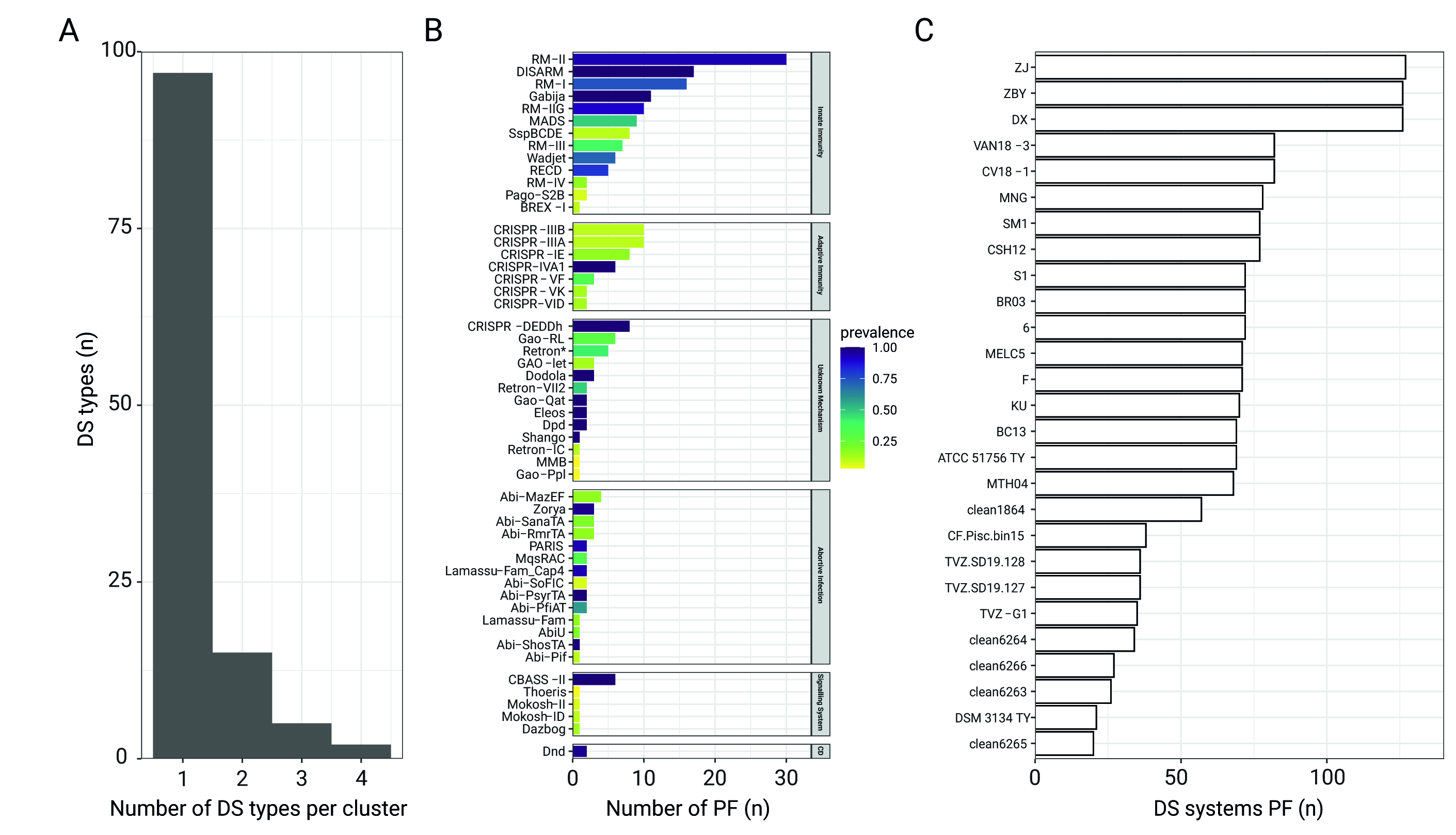

Supplement: Supplementary Figure S3 — Defense systems diversity and distribution in ‘F. caldus’ genomes. (A) Frequency histogram showing the number of defense systems (DS) that could be assigned to a single genomic locus, and therefore clustered as “defense islands”. (B) Number of DS-associated unique protein families (PFs), and their prevalence in ‘F. caldus’ genomes. The prevalence was defined as the number of genomes in which a given DS PF was identified divided by the total genomes analyzed. (C) Total number of DS-associated PFs identified in each ‘F. caldus’ genome sequence, ranked from highest to lowest. PFs were defined by clustering orthologs using ProteinOrtho (Lechner et al., 2011) with thresholds of 60% identity and 60% coverage. Defense systems were classified after (Tesson et al., 2022; Payne et al., 2022; Bernheim and Sorek, 2020; Millman et al., 2022; Doron et al., 2018). [file Image_3.TIF]

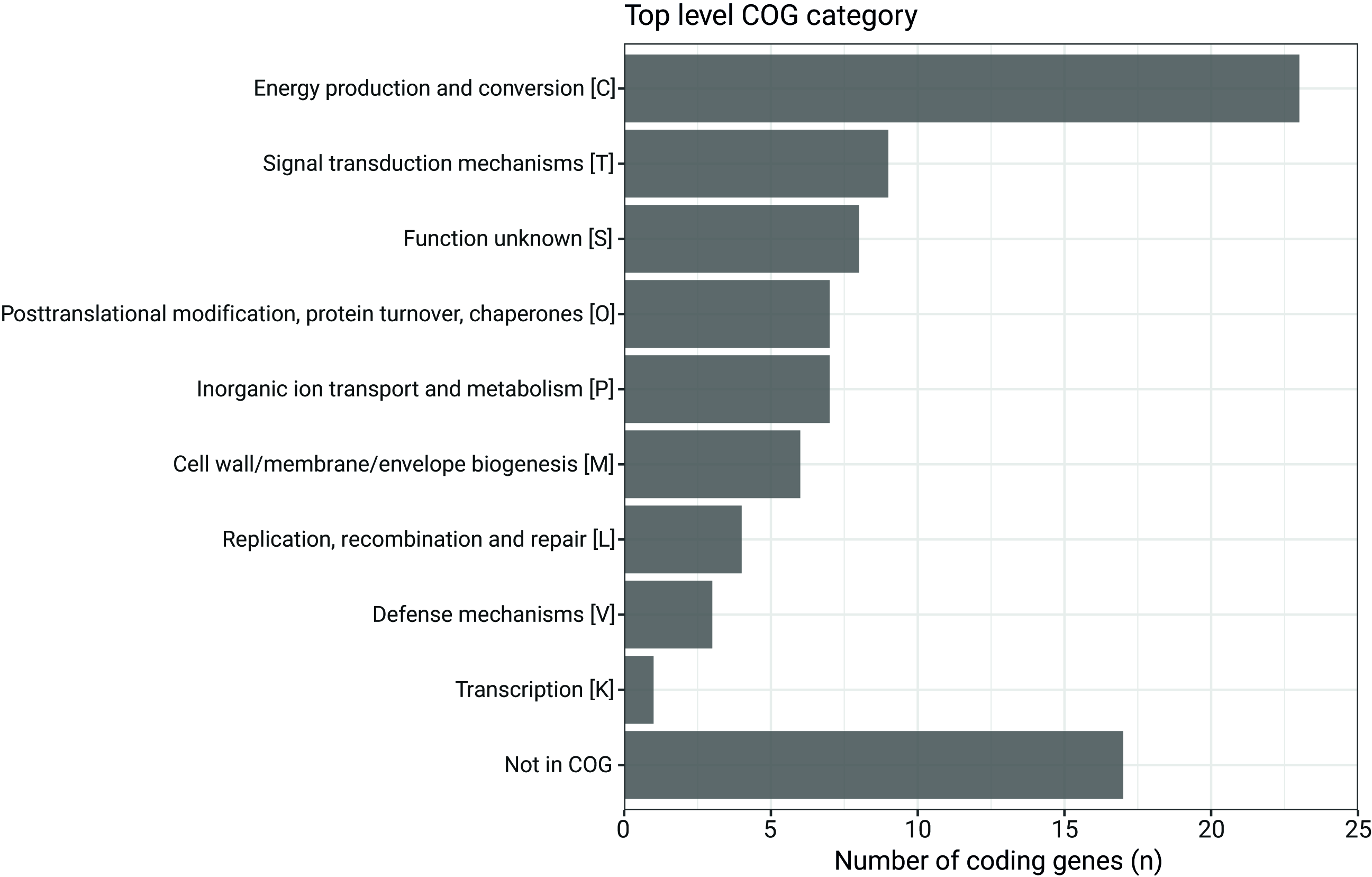

Supplement: Supplementary Figure S4 — Functional assignment of ‘F. caldus’ plasmid encoded protein cargo. Protein sequences recovered from non-backbone plasmid regions were used as queries for homology searches using the COG database by using eggNOG. Number of coding sequences assigned to a given COG top level categories. [file Image_4.TIF]
